# ROS-mediated activation and mitochondrial translocation of CaMKII contributes to Drp1-dependent mitochondrial fission and apoptosis in triple-negative breast cancer cells by isorhamnetin and chloroquine

**DOI:** 10.1186/s13046-019-1201-4

**Published:** 2019-05-28

**Authors:** Jinjiao Hu, Yanhao Zhang, Xiuxing Jiang, Hongwei Zhang, Ziyi Gao, Yunong Li, Ruoqiu Fu, Lirong Li, Jie Li, Hongjuan Cui, Ning Gao

**Affiliations:** 1 College of Pharmacy, Army Medical University, 30 Gaotanyan Street, Shapingba District, Chongqing, 400038 China; 2 Key Laboratory of Basic Pharmacology of Ministry of Education and Joint International Research Laboratory of Ethnomedicine of Ministry of Education, Zunyi Medical University, Zunyi, China; 3 Greater Philadelphia Pharmacy, Philadelphia, USA; 4 State Key Laboratory of Silkworm Genome Biology, Southwest University, 2#Tiansheng Road, Beibei District, Chongqing, 400716 China; 5 Medical Research Institute, Southwest University, 2#Tiansheng Road, Beibei District, Chongqing, 400716 China

**Keywords:** Triple-negative breast cancer, Chloroquine, Isorhamnetin, Autophagy, Apoptosis, CaMKII, Drp1

## Abstract

**Background:**

Triple-negative breast cancer (TNBC) is often aggressive and associated with a poor prognosis. Due to the lack of available targeted therapies and to problems of resistance with conventional chemotherapeutic agents, finding new treatments for TNBC remains a challenge and a better therapeutic strategy is urgently required.

**Methods:**

TNBC cells and xenograft mice were treated with a combination of chloroquine (CQ) and isorhamnetin (IH). Mitochondrial fission, apoptosis, and related signaling pathways were determined by flow cytometry, immunofluorescence, and related molecular biological techniques.

**Results:**

The inhibition of autophagy/mitophagy by CQ selectively enhances IH-induced mitochondrial fission and apoptosis in TNBC cells but not in estrogen-dependent breast cancer cells. These events were accompanied by mitochondrial translocation of Bax and the release of cytochrome c. Mechanistically, these effects were associated with oxidative stress-mediated phosphorylation of CaMKII (Thr286) and Drp1 (S616), and subsequent mitochondrial translocation of CaMKII and Drp1. The interruption of the CaMKII pathway by genetic approaches (e.g. CaMKII mutant or siRNA) attenuated combination-mediated mitochondrial fission and apoptosis. The combination of CQ/IH was a marked inhibitor tumor growth, inducing apoptosis in the TNBC xenograft mouse model in association with the activation of CaMKII and Drp1 (S616).

**Conclusions:**

Our study highlights the critical role of ROS-mediating CaMKII/Drp1 signaling in the regulation of mitochondrial fission and apoptosis induced by combination of CQ/IH. These findings also suggest that IH could potentially be further developed as a novel chemotherapeutic agent. Furthermore, a combination of IH with classic autophagy/mitophagy inhibitor could represent a novel therapeutic strategy for the treatment of TNBC.

**Electronic supplementary material:**

The online version of this article (10.1186/s13046-019-1201-4) contains supplementary material, which is available to authorized users.

## Background

Breast cancer is the most common malignancy and is a leading cause of cancer-related deaths in women worldwide [1]. Among its different subtypes, triple-negative breast cancer (TNBC) accounts for 15–20% of diagnosed breast tumors, there being a higher incidence in young and African-American women [2, 3]. TNBC constitutes a heterogeneous group of malignancies that are often aggressive and associated with a poor prognosis [4]. Due to a lack of available targeted therapies and to problems of resistance to conventional chemotherapeutic agents, finding new treatments for TNBC remains a challenge; a better therapeutic strategy is urgently required [5, 6].

Autophagy is a mechanism by which cellular material is delivered to lysosomes for degradation [7]. Autophagy acts as pro-survival pathway in cancer cells by promoting the viability and growth of these cells and imparts resistance in them to many chemotherapeutic agents in tumor cells, including TNBC cells [8]. Therefore, autophagy inhibition has been regarded as a promising therapeutic approach in the treatment of TNBC and other cancers. Recent evidence reveals that the inhibition of autophagy has been identified as a potential adjunctive strategy for enhancing the chemotherapeutic effect [9]. The antimalaria drugs chloroquine (CQ) and hydroxychloroquine (HCQ) are currently the only clinically-available drugs that inhibit autophagy. Extensive preclinical evidence reveals that the inhibition of autophagy by CQ or HCQ increases the potential of anticancer chemotherapeutic agents such as the antiretroviral Nelfinavir and the COX2 inhibitor Celecoxib in TNBC [10]. The current study reveals that the inhibition of autophagy by CQ could enhance the effects of chemotherapeutic agents in treating TNBC patients with high LC3B protein levels [11]. Owing to the limitations of current chemotherapeutic agents in the treatment of TNBC, it is important to develop novel and efficacious chemotherapeutic agents that target TNBCs.

**Fig. 1:**
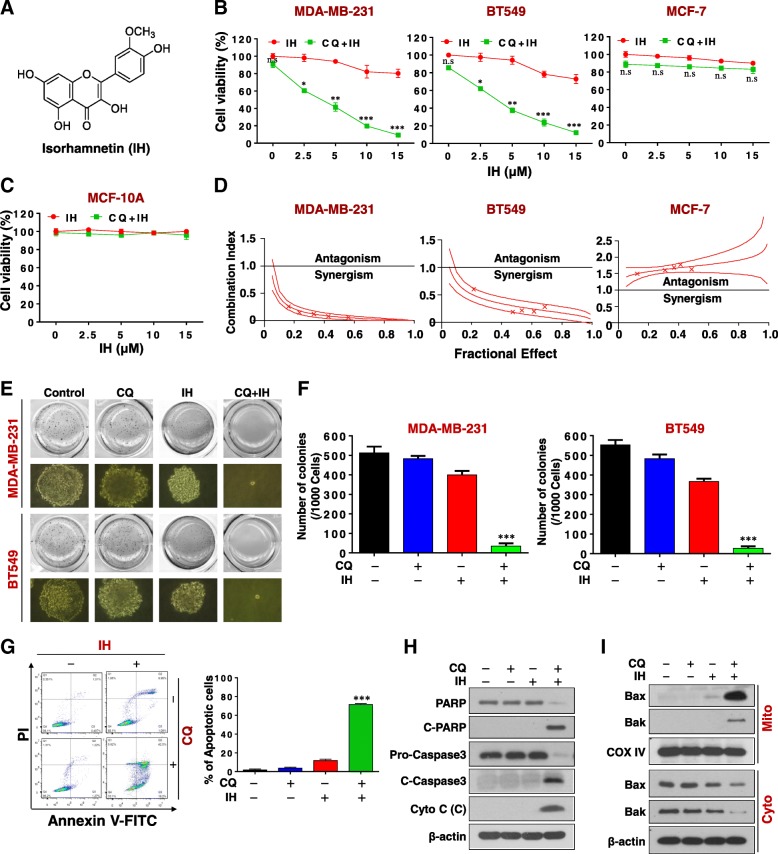
CQ dramatically potentiates IH-mediated inhibition of cell proliferation and the induction of apoptosis in TNBC cells. **a** The chemical structure of isorhamnetin (IH). **b** and **c** MDA-MB-231, BT549, MCF-7, and MCF-10A cells were treated with various concentrations of IH in the presence or absence of 20 μM CQ for 48 h, and MTT assays were performed to assess cell proliferation—mean ± SD for three independent experiments, ns, not significant, ^*^P < 0.05, ^**^P < 0.01 or ^***^P < 0.001 compared with IH. **d** The combination index (CI) values for each fraction affected were determined using commercially-available software (Calcusyn, Biosoft). CI values less than 1.0 correspond to synergistic interactions. **e** and **f** Colony formation was detected using a soft agar assay in MDA-MB-231 and BT549 cells (mean ± SD for three independent experiments, ^***^P < 0.001 compared with control). **g**-**i** MDA-MB-231 cells were combination treated with CQ (20 μM) and IH (10 μM) for 48 h. Apoptosis was determined by Annexin V-FITC/PI staining and flow cytometry (mean ± SD for 3 independent experiments; ^***^P < 0.001 compared with control or CQ and IH treatment alone). The total cellular extract, cytosol and mitochondrial fractions were prepared and subjected to western blot using antibodies against total PRAP, C-PARP, pro-Caspase 3, cleaved caspase-3, cytochrome c (Cyto C), Bak, and Bax. β-actin and COX IV were used as loading controls

Isorhamnetin (IH), also called 3-O-methylquercetin (Fig. 1a), is a flavonoid that is present in plants of the *Polygonaceae* family; it is also an immediate metabolite of quercetin in mammals [12]. IH has received attention due to its antitumor properties in cancers such as lung, esophageal, gastric, colorectal, skin, and breast cancers [13–18]. IH has displayed a diversity of anti-tumor activities, including inhibiting migration and invasion, inhibiting cell proliferation, and the induction of apoptosis through various signaling pathways (e.g. p38/STAT3, MEK, Akt/mTOR). It has recently been shown that IH induces autophagy in human breast cancer cells through modulating the PI3K/AKT/mTOR/p70S6K/ULK signaling pathway [19]. Yuan Y, et al. reported that the inhibition of autophagy by CQ enhances IH-induced mitochondria-dependent apoptosis in non-small lung cancer cells. However, the precise mechanism by which the inhibition of autophagy potentiates IH-induced mitochondrial apoptosis in breast cancer cells remains unclear.

In this study, we discovered that the inhibition of autophagy/mitophagy by CQ selectively enhances IH-induced mitochondrial fission and apoptosis in TNBC cells but not in estrogen-dependent breast cancer cells. Mechanistically, this effect is mediated by oxidative stress-phosphorylated Ca^2+^/calmodulin-dependent kinase II (CaMKII) (Thr286) and Drp1 (S616) and, subsequently, their mitochondrial translocation. Our data identify autophagy as a novel prognostic marker for TNBC: a combination of IH with CQ could represent a novel therapeutic strategy for treating TNBC.

## Material and methods

### Chemicals and antibodies

Isorhamnetin was purchased from Must Biotechnology (Chengdu, China); chloroquine from Sigma-Aldrich; Mn-TBAP from Focus Biomolecules; apocynin was purchased from Selleck Chemicals (Shanghai, CA). The antibodies against cleaved-caspase 3 (9661), pro-caspase 3 (9668S), p62 (5114S), phospho-CamkII (T286, 12,716), phospho-Drp1 (S616, 3455), phospho-Drp1 (S637, 4876), and Drp1 (8570) were purchased from Cell Signaling Technology; PARP (1078–1) was purchased from Epitomics; β-actin (A1978) and LC3 (L754S) from Sigma-Aldrich; Bax (510804), Cleaved-PARP (380374), Bak (380976), NOX2 (381293), NOX4 (380874) and COX IV (200147) from Zen-Bio. Cytochrome. C (13156) and CamkII (5306) were purchased Santa Cruz Biotechnology.

### Cell culture

MDA-MB-231 and MCF-7 cells were cultured in DMEM medium; BT549 cells in RPMI 1640 medium; MCF-10A in MEBM medium. All media comprised 10% fetal bovine serum (FBS) and antibiotics. All cell lines were obtained from the American Type Culture Collection (ATCC, Manassas, VA) and cultured at 37 °C in a humidified atmosphere with 5% CO_2_.

### Cell viability (MTT) assay

Cells were seeded in 96 well plates (3.5 × 10^3^ per each well). After treatment, 20 μl MTT (5 mg/ml) was added in each well and incubated at 37 °C for 4 h. After the medium was discarded, each well was supplemented with 150 μl DMSO to dissolve the formazan before being measured by a microplate reader at 490 nm. The cell viabilities were normalized to the control group.

### Soft agar assay

Sustainment gel was mixed with 0.6% agarose (Sigma-Aldrich) in a cell culture medium in 12 well plates. One-thousand cells were cultured in cultivate gel above concretionary sustainment gel (mixed with 0.3% agarose in cell culture medium with 10% FBS). After 28 days, the colonies were counted and photographed.

### Apoptosis assay

Apoptosis was examined by flow cytometry according to the manufacturer’s instructions (BD Biosciences PharMingen). In summary, 1 × 10^6^ cells were washed twice with phosphate-buffered saline (PBS) and stained with 5 μl Annexin V-FITC and 10 μl PI for 15 min at room temperature in the dark. Quantification of apoptotic cells was performed by flow cytometry using a FACScan cytofluorometer (BD Biosciences). Both early (Annexin V-positive, PI-negative) and late (Annexin V-positive and PI-positive) apoptotic cells were included in the cell death determinations.

### Mitochondrial and cytosolic fractionation

Mitochondrial and cytosolic fractionation were obtained as previously described [20]. In summary, cell pellets were washed twice with PBS and resuspended in 5 × Buffer A (20 mM HEPES, 10 mM KCl, 1.5 mM MgCl_2_, 1 mM EDTA, 1 mM EGTA, 1 mM Na_3_VO_4_). Cells were homogenized by passing them 15 times through a 22-gauge needle. The homogenate was centrifuged at 1000 g at 4 °C for 10 min. The supernatant was then transferred and continued being centrifuged at 3500 g at 4 °C for 10 min. The pellet fraction was considered the “mitochondrial” fraction. The supernatant fraction was then centrifuged at 120000 g at 4 °C for 10 min; the supernatant fraction was then considered the “cytosolic” fraction.

### Western blot and immunoprecipitation

The protein samples (30–50 μg) were separated using SDS-PAGE and transferred to PVDF membranes (Bio-Rad, 162–0177). After blocking with 5% fat-free dry milk in 1 × Tris-buffered saline (TBS), the membrane was probed overnight with primary antibodies at 4 °C. Protein bands were detected by incubating with horseradish peroxidase-conjugated antibodies (Kirkegaard and Perry Laboratories, Gaithersburg, MD, USA) and visualized with enhanced chemiluminescence reagent (Perkin-Elmer, Boston, MA, USA). For immunoprecipitation analysis, equal quantities of proteins were incubated with primary antibodies at 4 °C on a rocking platform. Immune complexes were collected with protein A/G agarose beads (Beyotime Technology), washed in PBS five times, and subjected to Western blot.

### Detection of calcium ion level

The Ca^2+^ level was determined by using the fluorescent dye Fluo-4/AM (Invitrogen). Briefly, cells were washed three times with HBSS, then incubated with 4 μM Fluo-4/AM (diluted with HBSS) at 37 °C for 30 min. After being washed twice with HBSS, followed by an additional 15-min incubation at 37 °C to allow complete de-esterification of intracellular AM esters, the cells were detected by flow cytometry using a FACScan cytofluorometer (BD Biosciences).

### Immunofluorescence

Cells were seeded on coverslips and cultured in 24 well plates for 24 h. After treatment for 24 h, mitochondria were stained with MitoTracker Deep Red FM (Molecular Probes, Carlsbad, USA) according to the manufacturer’s instructions. Cells were fixed with 4% formaldehyde (Beyotime Biotechnology) for 30 min, permeabilized with 0.1% Triton X-100 in PBS for 5 min, and then blocked with goat serum (Beyotime Biotechnology) in PBS for 30 min. The cells were incubated overnight with primary antibodies at 4 °C, followed by the appropriate secondary antibodies at 37 °C for 1 h. The cells were viewed using a laser-scanning confocal microscope (Zeiss, Germany). All images were analyzed by ImageJ software (MD, USA).

### Detection of intracellular ROS

Intracellular production of ROS was measured using DCFH-DA. To determine ROS production, cells were incubated with DCFH-DA (10 μM) for 30 min, washed twice with cold PBS and detected by flow cytometry using a FACScan cytofluorometer (BD Biosciences).

### RNA interference and site mutant

The target sequence of CamkII shRNA (5′-CGTAAATGGATTTCGCGTTAA-3′) was constructed by Gene Chem Co. Ltd. (Shanghai, China). To generate CamkII knockdown stable cell lines, a lentiviral system was employed as previously described [21]. Briefly, 293FT cells were co-transfected with lentiviral packing vectors pLP1, pLP2 and pLP/VSVG (Invitrogen), along with shCamkII or shCon plasmid, using Lipofectamine 3000 (Invitrogen) for 48 h. The supernatant containing the lentivirus was harvested and used for infection with MDA-MB-231 cells. The cells were subsequently selected with 8 μg/mL puromycin to establish stable cell lines. Site mutant plasmids of CamkII (CamkII^T286A^ and CamkII^T286D^) were constructed by Gene Chem Co. Ltd. (Shanghai, China). The sequence of primers for CamkII^T286A^ were forward 5′-CACAGACAGGAGGCCGTGGACTGCCTG-3′, and reverse 3′-GTGTCTGTCCTCCGGCACCTGACGGAC-5′. Primers for CamkII^T286D^ were forward 5′-CACAGACAGGAGGATGTGGACTGCCTG-3′, and reverse 3′-GTGTCTGTCCTCCTACACCTGACGGAC-5′. MDA-MB-231 cells were transfected with CamkII^T286A^ and CamkII^T286D^ using Lipofectamine 3000 according to the manufacturer’s instructions.

### Xenograft assay

Female nude mice (5–6 weeks old) were purchased from Vital River Laboratories (VRL, Beijing, China) and fed a standard animal diet and water. The animal studies were approved by the University Institutional Animal Care and Use Committee. MDA-MB-231 cells were suspended in a 1:1 ratio in DMEM medium with a Matrigel basement membrane matrix (Sigma, E1270). Cells (4 × 10^7^) were inoculated in the right legs of mice. After tumor inoculation, the mice were randomly divided into four treatment groups (16 mice per group; six mice were used for body weight and tumor volume measurement, the others for survival analysis). The mice were treated with either vehicle, CQ (40 mg/kg) or IH (20 mg/kg), or a combination of CQ/IH by intraperitoneal injection once every 2 days. The body weight and tumor volume (mm^3^) were measured. The mice were euthanized 30 days after medication, the tumors were excised and were either formalin-fixed or flash-frozen at − 20 °C. H&E, TUNEL, and immunohistochemical analyses were performed as previously described [22].

### Statistical analysis

All data values are represented as mean ± SD. The comparisons were performed using Student’s t-test or one-way analysis of variance (ANOVA). Survival analysis in vivo was performed using the Kaplan–Meier method and significance was calculated using the log-rank test. ^*^P < 0.05, ^**^P < 0.01, and ^***^P < 0.001 were regarded as significant differences.

## Results

### Chloroquine dramatically potentiates isorhamnetin-mediated inhibition of cell proliferation and induction of apoptosis in triple negative breast cancer cells

The effects of combined treatment with CQ and IH on cell viability were first investigated in multiple human breast cancer cell lines. Exposure to a subtoxic concentration of CQ (20 μM) significantly decreased the cell viability in both triple negative breast cancer MDA-MB-231 and BT549 cells treated with a nontoxic concentration of IH (2.5 μM), and the degree of potentiation increased as concentrations increased (Fig. 1b). In contrast, CQ in combination with IH exerted little effect on cell viability toward MCF-7 (estrogen-dependent) cells (Fig. 1b) and normal breast epithelial MCF-10A cells (Fig. 1c). The median dose effect analysis of cell viability in cells exposed to CQ and IH for 48 h at fixed ratios yielded CI values consistently less than 1.0 in MDA-MB-231 and BT549 cells but greater than 1.0 in MCF-7 cells (Fig. 1d). We also examined the effects of CQ/IH on colony formation in both MDA-MB-231 and BT549 cells in vitro by using soft agar assay. As shown in Fig. 1e and f, the combination of CQ/IH significantly decreased the number of colonies in MDA-MB-231 and BT549 cells. These results indicate that the combination of CQ/IH selectively inhibits cell proliferation and tumorigenesis in TNBC cells.

We next investigated the synergistic effects of CQ/IH on apoptosis in MDA-MB-231 and BT549 cells. Combined treatment with minimally-toxic concentrations of CQ (20 μM) and IH (10 μm) resulted in a pronounced increase in apoptosis in MDA-MB-231 and BT549 cells (Fig. 1g and Additional file 1: Figure S1A). Consistent with these findings, the same CQ and IH concentrations resulted in a degradation of PARP, cleavage/activation of caspases-3 and release of cytochrome c into the cytosolic fraction (Figs. 1h and Additional file 1: Figure S1B). The translocation of Bax and Bak from the cytosol to the mitochondria was also noted in cells treated with a combination of CQ/IH (Fig. 1i and Additional file 1: Figure S1C). Together, these findings indicate that CQ interacts synergistically with IH to selectively induce mitochondrial injury and apoptosis in TNBC cells.

### Excessive accumulation of mitophagosomes contributes to mitochondrial injury and apoptosis mediated by a combination of CQ and IH

**Fig. 2:**
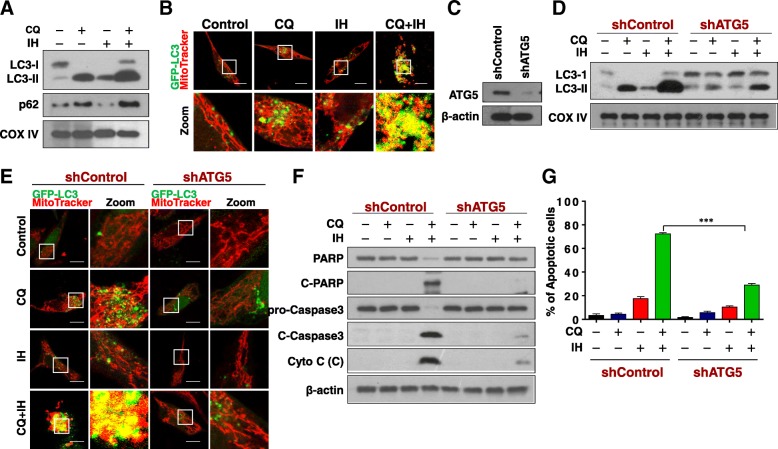
Excessive accumulation of mitophagosomes contributes to apoptosis induced by CQ/IH combination. **a** MDA-MB-231 cells were treated with CQ (20 μM) in the presence or absence of IH (10 μM) for 48 h, after which the mitochondrial fractions were prepared and subjected to Western blot analysis using antibodies against p62, LC3-I/LC3-II. **b** Cells expressed with EGFP-LC3 were treated with CQ (20 μM) in the presence or absence of IH (10 μM) for 48 h. The colocalization of EGFP-LC3 and MitoTracker (Deep Red FM) was examined using confocal microscopy. Scale bars: 10 μm. **c** Cells were transfected with control shRNA (shControl) or shATG5, and Western blot analysis was used to determine the expression of ATG5. For **d**-**g**, cells stably expressing shControl or shATG5 were treated with CQ (20 μM) in the presence or absence of IH (10 μM) for 48 h. **d** The mitochondrial fractions were prepared and subjected to Western blot using antibodies against LC3-I/LC3-II. **e** The colocalization of EGFP-LC3 and MitoTracker (Deep Red FM) was examined by confocal microscopy. Scale bars: 10 μm. **f** The expressions of total PRAP, C-PARP, pro-Caspase 3, C-Caspase 3 and Cyto C (Cytosolic fraction) were determined by Western blot. **g** Apoptosis was detected by flow cytometry analysis. The values obtained from the Annexin V/PI assay represent the mean ± SD for three separate experiments. ^***^Values for cells combination-treated with CQ/IH after transfection with shATG5 are significantly decreased compared with those in shControl cells combined treated with CQ/IH, ^***^P < 0.001

Since CQ suppresses autophagic flux by blocking autophagosome-lysosome fusion, we subsequently investigated the effects of the combination of CQ/IH on the accumulation of mitophagosomes. As shown in Fig. 2a, treating cells with CQ alone resulted in the accumulation of LC3B-II and p62 in mitochondria. Treating cells with IH, an autophagy inducer [19], resulted in modest increases in levels of LC3B-II and decreases in levels of p62 in mitochondria. Joint treatment with CQ/IH resulted in excessive accumulation of LC3B-II and p62 in mitochondria. Similarly, significant increases in colocalization of GFP-LC3 and RFP-Mito were observed in cells combined-treated with CQ/IH (Fig. 2b), suggesting that excessive accumulation of mitophagosomes may be involved in mitochondrial injury and apoptosis in cells treated with a combination of CQ/IH. To test this possibility, a siRNA approach was used to stably knock down ATG5 expression (Fig. 2c). Knockdown of ATG5 markedly reduced combination-mediated LC3B-II accumulation in mitochondria (Fig. 2d) and mitophagosome formation (Fig. 2e). Knockdown of ATG5 also abrogated combination-mediated degradation of PARP, cleavage/activation of caspase-3 and cytochrome c release (Fig. 2f), as well as apoptosis (Fig. 2g and Additional file 1: Figure S2). Together, these findings indicate that the excessive accumulation of mitophagosomes is implicated in mitochondrial injury and apoptosis mediated by the combination of CQ/IH in TNBC cells.

### The combination of CQ/IH induces mitochondrial fission through phosphorylation of CamkII (Thr286) and Drp1 (Ser616) and their mitochondrial translocation

**Fig. 3:**
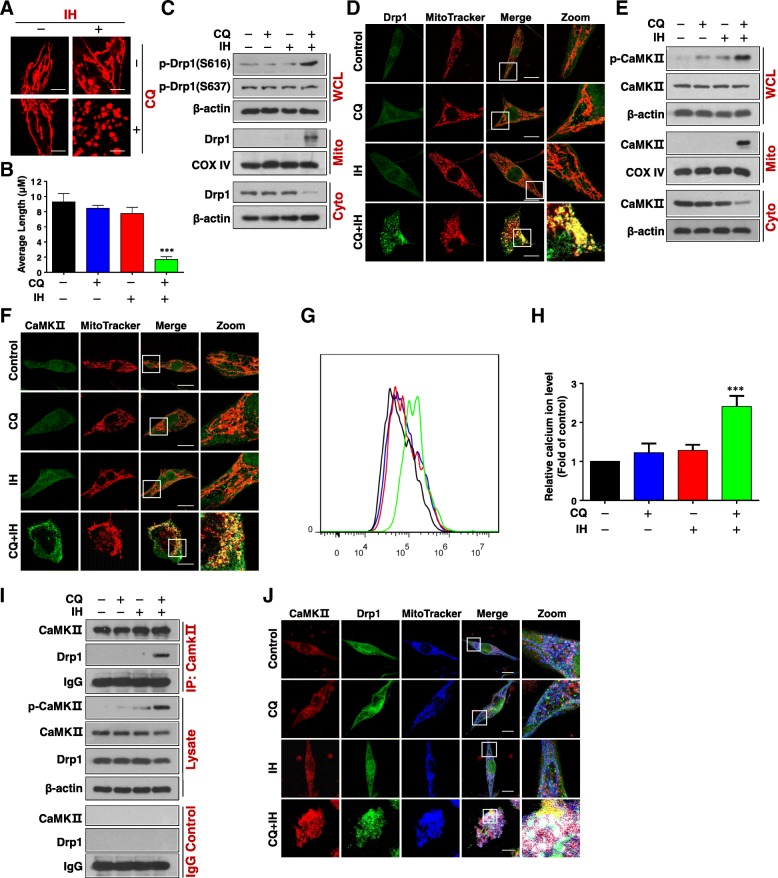
Combination of CQ/IH induces phosphorylation and mitochondrial translocation of CaMKII (Thr286) and Drp1 (Ser616). MDA-MB-231 cells were treated with CQ (20 μM) in the presence or absence of IH (10 μM) for 48 h. **a** Mitochondrial morphology was observed by MitoTracker Red CMXRos staining and confocal microscopy. Scale bars: 10 μm. **b** Mitochondrial length was measured with ImageJ software. 50 cells from three independent experiments (mean ± SD, ^***^P < 0.001 compared with control). **c** Whole cellular lysates (WCL) and cytosolic (Cyto)/mitochondrial (Mito) fractions were prepared and subjected to Western blot using antibodies against phospho-Drp1 (p-Drp1) (S616), p-Drp1 (S637), and Drp1. **d** The colocalization of Drp1 (green) and MitoTracker (red) was examined using confocal microscopy. Scale bars: 10 μm. **e** The expression of p-CaMKII and CaMKII in WCL, Cyto, or Mito was examined by Western blot. **f** The colocalization of CaMKII (green) and MitoTracker (red) was examined by confocal microscopy. Scale bars: 10 μm. **g** and **h** The calcium ion level was analyzed by flow cytometry. The values represent the mean ± SD for three separate experiments (^***^P < 0.001 compared with control). **i** Whole cell lysates were prepared and subjected to immunoprecipitation using anti-CaMKII; the associated CaMKII and Drp1 were determined using immunoblotting. **j** The colocalization of CaMKII (red), Drp1 (green), and MitoTracker (blue) was examined using confocal microscopy. Scale bars: 10 μm

Recent studies indicate that mitochondrial fission participates in Bax-mediated permeabilization of the outer mitochondrial membrane and cytochrome c release [23]. Therefore, we next examined the effects of the combination of CQ/IH on mitochondrial dynamics using MitoTracker Red CMXRos. The combination of CQ/IH resulted in a significant increase in the proportion of cells with fragmented mitochondria (Fig. 3a and b, Additional file 1: Figure S3A and S3B).

Increasing evidence reveals that phosphorylation of Drp1 (Ser 616) or dephosphorylation of Drp1 (Ser 637) plays a critical role in the regulation of mitochondrial fission through its mitochondrial translocation [24]. We then examined the effects of the combination of CQ/IH on the phosphorylation of Drp1 at Ser637 and Ser616, and the mitochondrial translocation of Drp1. Combined treatment with CQ/IH increased levels of phospho-Drp1 (Ser 616) but had no effect on the phosphorylation of Drp1 at Ser 637 (Fig. 3c, Additional file 1: Figure S3C). The combination of CQ/IH also led to the mitochondrial translocation of Drp1 (Fig. 3c, Additional file 1: Figure S3C). Immunofluorescence analysis showed the Drp1 signal in the mitochondria of cells treated with a combination of CQ/IH (Fig. 3d, Additional file 1: Figure S3D). Since the colocalization of Drp1 and Bax at the mitochondrial fission site is required for mitochondrial fission and apoptosis [25], we next examined the effect of a combination of CQ/IH on the colocalization of Drp1 and Bax in mitochondria by using immunofluorescence analysis. The colocalization of Drp1 and Bax in mitochondria was observed in cells treated with a CQ/IH combination (Additional file 1: Figure S4). These results support the proposition that the phosphorylation of Drp1 at the S616 site promotes its mitochondrial translocation, leading to mitochondrial fission and apoptosis through the colocalization of Drp1 and Bax in response to a combination of CQ/IH.

Recent evidence reveals that the phosphorylation of Drp1 at S616 site promotes its mitochondrial translocation upon activation by Ca^2+^/calmodulin-dependent kinase II (CaMKII) [26]. We next examined the effects of the CQ/IH combination on the phosphorylation of CaMKII (Thr286). Exposure to the CQ/IH combination resulted in marked increases in levels of phosphor-CaMKII (Thr286). However, this increase was not present when the agents were administered individually (Fig. 3e, Additional file 1: Figure S5A). Interestingly, combined treatment with CQ/IH led to a decrease in the levels of CaMKII in cytosol and increased levels of CaMKII in mitochondria (Fig. 3e, Additional file 1: Figure S5A). Similarly, immunofluorescence analysis showed the CaMKII signal in the mitochondria in the cells that were treated with a combination of CQ/IH (Fig. 3f, Additional file 1: Figure S5B). Such findings suggest that the phosphorylation of CaMKII promotes its mitochondrial translocation in response to a combination of CQ/IH.

Since CaMKII was regulated by calcium ions [27], we next determined the levels of intracellular calcium ions by using the fluorescent calcium indicator Fluo-4/AM. As shown in Fig. 3g and h, the combination of CQ/IH significantly increased the levels of intracellular calcium ions in MDA-MB-231 cells.

Since phosphorylation of Drp1 (S616) can be modulated by kinases/phosphatases including CaMKII, it is critical to determine whether CaMKII can directly bind Drp1. Immunoprecipitation analysis showed that combined treatment with CQ/IH resulted in increased interaction of CaMKII and Drp1 (Fig. 3i, Additional file 1: Figure S5C). Immunofluorescence analysis also showed the colocalization of MitoTracker with CaMKII and Drp1 in cells treated with a combination of CQ/IH (Fig. 3j, Additional file 1: Figure S5D). Taken together, these findings suggest that a combination of CQ/IH induces mitochondrial fission through the activation of CaMKII and its mitochondrial translocation, leading to the phosphorylation and mitochondrial translocation of Drp1.

### Genetic interruption of CaMKII abrogates mitochondrial fission and apoptosis induced by CQ/IH

**Fig. 4:**
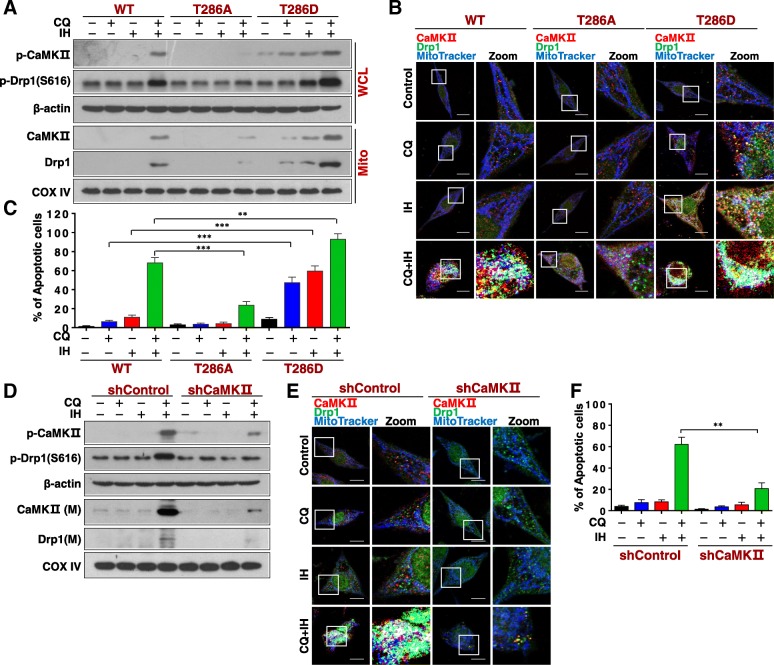
Genetic interruption of CaMKII blocks mitochondrial fission and apoptosis induced by CQ/IH combination. MDA-MB-231 cells transfected with either wild type CaMKII (WT) or mutants CaMKII^T286A^ (T286A) or CaMKII^T286D^ (T286D) were treated with CQ (20 μM) in the presence or absence of IH (10 μM) for 48 h. **a** The expressions of p-CaMKII in WCL, and CaMKII and Drp1 in Mito were examined by Western blot. **b** The colocalization of CaMKII (red), Drp1 (green), and MitoTracker (blue) was examined by confocal microscopy. Scale bars: 10 μm. **c** Apoptosis was detected by flow cytometry analysis. The values represent the mean ± SD for three separate experiments (^**^P < 0.01, ^***^P < 0.001). For **d**–**f**, cells transfected with either shControl or shCaMKII were treated with CQ (20 μM) in the presence or absence of IH (10 μM) for 24 h. **d** The expressions of p-CaMKII in WCL, and of CaMKII and Drp1 in Mito were examined by Western blot. **e** The colocalization of CaMKII (red), Drp1 (green), and MitoTracker (blue) was examined by confocal microscopy. Scale bars: 10 μm. **f** Apoptosis was detected by flow cytometry analysis. The values represent the mean ± SD for three separate experiments (^**^P < 0.01)

In order to further investigate the role of CaMKII phosphorylation at Thr286 in mitochondrial fission and apoptosis induced by the combination of CQ/IH, we generated a mutant of CaMKII^T286A^ to occlude Thr286 phosphorylation or a mutant of CaMKII^T286D^ to mimic Thr286 phosphorylation (Fig. 4a). Overexpression of CaMKII^T286A^ blocked the phosphorylation of CaMKII (Thr286)/Drp1 (S616) and the mitochondrial translocation of CaMKII/Drp1 in cells treated with a combination of CQ/IH. On the other hand, an overexpression of CaMKII^T286D^ promoted the phosphorylation of CaMKII (Thr286)/Drp1 (S616) and the mitochondrial translocation of CaMKII/Drp1 in cells treated with either CQ or IH alone or a combination of these (Fig. 4a). CaMKII^T286A^ also blocked the colocalization of MitoTracker with CaMKII and Drp1 in cells treated with combination, whereas CaMKII^T286D^ increased the colocalization of MitoTracker with CaMKII and Drp1 in cells treated with either CQ or IH alone or a combination of these (Fig. 4b). Furthermore, CaMKII^T286A^ attenuated mitochondrial fission, the mitochondrial translocation of Bax, the activation of caspase-3, the release of cytochrome c, and apoptosis induced by combination, whereas CaMKII^T286D^ promoted these events mediated by either CQ or IH alone or their combination (Fig. 4c, Additional file 1: Figure S6A-S6D). Thus, our data indicate that the phosphorylation and mitochondrial translocation of CaMKII play critical roles in mitochondrial fission and apoptosis induced by a combination of CQ/IH.

To further investigate the functional significance of CaMKII activation in CQ/IH-mediated mitochondrial fission and apoptosis, a siRNA approach was used to knock down CaMKII expression in a stable way (Additional file 1: Figure S7A). The knockdown of CaMKII blocked phosphorylation of CaMKII (T286)/Drp1 (S616) and mitochondrial translocation of CaMKII/Drp1 that was mediated by a combination of CQ/IH (Fig. 4d). This knockdown of CaMKII also blocked the colocalization of MitoTracker with CaMKII and Drp1 in cells treated with the combination (Fig. 4e). In addition, the knockdown of CaMKII blocked mitochondrial fission, the mitochondrial translocation of Bax, the activation of caspase-3, the release of cytochrome c, and apoptosis induced by combination (Fig. 4f, Additional file 1: Figure S7B-S7E). These results further identified the functional role of CaMKII in CQ/IH-mediated mitochondrial fission and apoptosis.

### Combined treatment with CQ/IH induces generation of reactive oxygen species

**Fig. 5:**
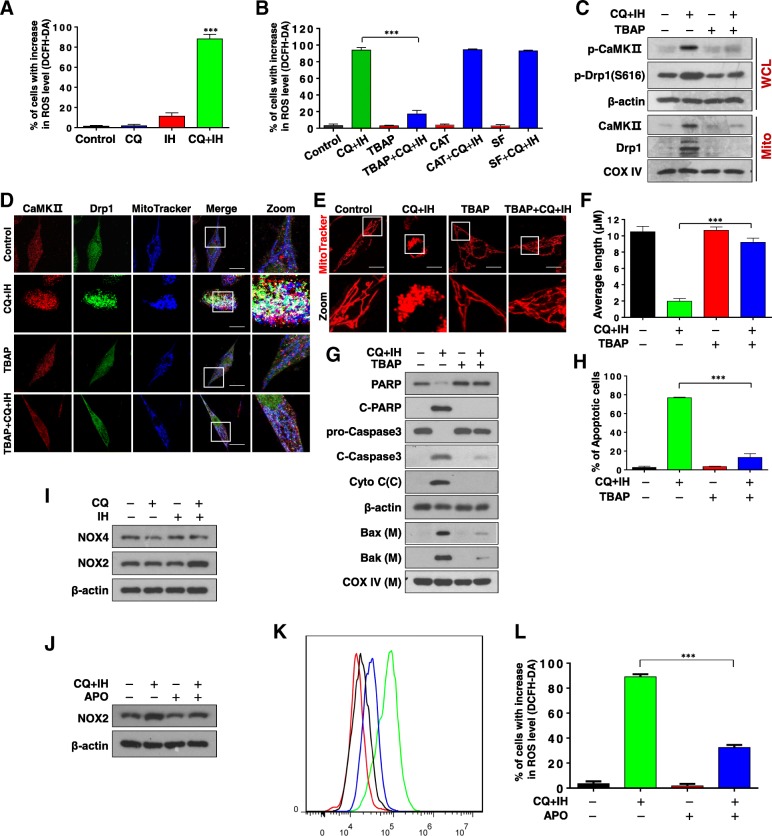
Effects of antioxidants on CQ/IH-induced ROS generation, mitochondrial fission, apoptosis, and cell signaling proteins. **a** MDA-MB-231 cells were treated with CQ (20 μM) in the presence or absence of IH (10 μM) for 6 h. Cells were stained with DCFHDA, and ROS production was analyzed by flow cytometry as described in Materials and Methods (mean ± SD for three independent experiments; ^***^P < 0.001 compared with control or CQ and IH treatment alone). **b** Cells were pretreated with antioxidants including TBAP (200 μM), catalase (5000 U/ml), and sodium formate (SF, 2 mM) for 1 h, followed by combined treatment with CQ/IH, after which ells were stained with DCFHDA; ROS production was then analyzed by flow cytometry (mean ± SD, ^***^P < 0.001). For C–H, cells were pretreated with TBAP, followed by the CQ/IH combination. **c** WCL and Mito were prepared and subjected to Western blot using antibodies against p-CaMKII (T286), p-Drp1 (S616), CaMKII, and Drp1. **d** The colocalization of CaMKII (red), Drp1 (green), and MitoTracker (blue) was examined by confocal microscopy. Scale bars: 10 μm. **e** Mitochondrial morphology was observed by MitoTracker Red CMXRos staining and confocal microscopy. Scale bars: 10 μm. **f** Mitochondrial length was measured with ImageJ software. Fifty cells of three independent experiments (mean ± SD, ^***^P < 0.001). **g** WCL, Cyto, and Mito were prepared and subjected to Western blot using antibodies against total PRAP, C-PARP, pro-Caspase 3, C-Caspase 3, cytochrome c, Bak, and Bax. **h** Apoptosis was detected by flow cytometry analysis. The values represent the mean ± SD for three separate experiments (mean ± SD, ^***^P < 0.001). **i** MDA-MB-231 cells were treated with CQ (20 μM) in the presence or absence of IH (10 μM) for 48 h. WCL were prepared and subjected to Western blot analysis using antibodies against NOX4 and NOX2, β-actin being used as a loading control. **j** Cells were pretreated with APO (100 μM) for 2 h, followed by the combination of CQ/IH. WCL were prepared and subjected to Western blot analysis using antibodies against NOX2, β-actin being used as a loading control. **k** and **l** Cells were pretreated with APO (100 μM) for 2 h, followed by the combination of CQ/IH for 6 h. Cells were stained with DCFHDA and ROS production was analyzed by flow cytometry. (mean ± SD for three independent experiments; ^***^P < 0.001)

Several types of evidence have demonstrated that activation of CaMKII is attributable to excessive production of reactive oxygen species (ROS) [28, 29]. We thus examined the effects of the CQ/IH combination on the generation of ROS in MDA-MB-231 and BT549 cells. By using flow cytometry analysis, we found that combined exposure of cells to CQ/IH resulted in significant increases in the generation of ROS (Fig. 5a, Additional file 1: Figure S8A). ROS—including superoxide radical (O_2_^•-^), hydrogen peroxide (H_2_O_2_), and hydroxyl radical (OH•)—are recognized as signaling molecules that are mobilized in response to various stimuli [30]. To explore further the role of individual ROS on combination-mediated mitochondrial fission and apoptosis, we employed three antioxidants—TBAP (a cell permeable SOD mimetic), catalase, and sodium formate—which primarily act on O_2_^•-^, H_2_O_2_, and OH•, respectively. Pretreatment with TBAP, an O_2_^•-^ scavenger, abrogated combination-mediated ROS generation in both MDA-MB-231 and BT549 cells. In contrast, catalase (a H_2_O_2_ scavenger) and sodium formate (an OH• scavenger) failed to block combination-mediated ROS generation in these cells (Fig. 5b, Additional file 1: Figure S8B). Attempts were then made to assess the functional significance of ROS in combination-induced mitochondrial fission and apoptosis. The addition of TBAP (but not of catalase and sodium formate) essentially abrogated the combination-mediated phosphorylation of CaMKII (T286)/Drp1 (S616) and the mitochondrial translocation of CaMKII/Drp1 (Fig. 5c). Pretreatment with TBAP also abrogated combination-mediated colocalization of MitoTracker with CaMKII and Drp1 (Fig. 5d). Furthermore, the addition of TBAP markedly abrogated combination-induced mitochondrial fission (Fig. 5e and f). Finally, the addition of TBAP significantly abrogated the combination-mediated mitochondrial translocation of Bax and Bak, the degradation of PARP, the activation of caspase 3, the release of cytochrome c, and apoptosis (Fig. 5g and h, Additional file 1: Figure S8C). Collectively, these finding suggest that ROS, particularly O_2_^•-^ radical, are primarily responsible for combination-induced mitochondrial fission and apoptosis through perturbations in CaMKII/Drp1 signaling events.

Increasing evidence indicates that NADPH oxidase (NOX) is a major source of superoxide anion radical (O_2_^•-^) generation, and that NOX2 and NOX4 are key enzymes responsible for the oxidative burst [31, 32]. Therefore, we next examined the effect of a combination of CQ/IH on the expression of NOX2 and NOX4. As shown in Fig. 5i, a CQ/IH combination increased the levels of NOX2 but had no effect on NOX4. In addition, pretreatment with apocynin, a selective NADPH oxidase inhibitor, abrogated combination-induced NOX2 expression and ROS generation (Fig. 5j, k and l).

### Inhibition of mitophagy enhances the inhibitory effect of IH on tumor growth in a TNBC xenograft mouse model in vivo

**Fig. 6:**
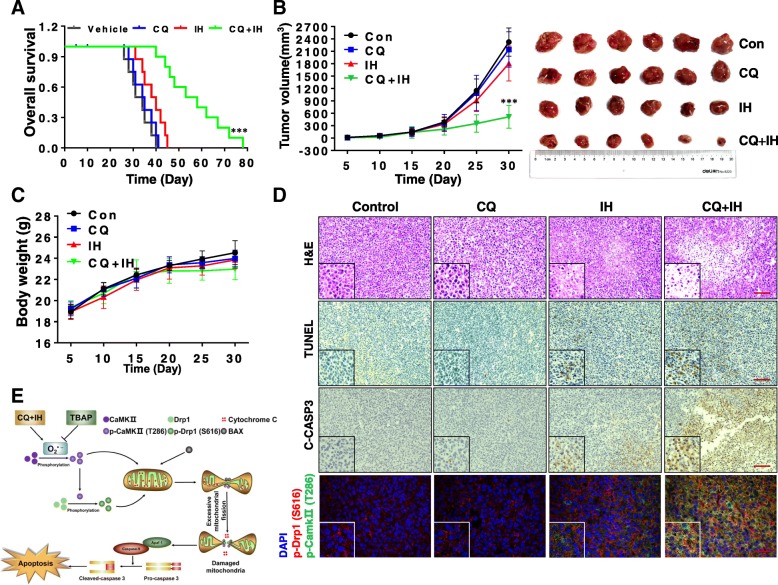
The combination of CQ/IH inhibits tumor growth in a TNBC xenograft mouse model. Sixty-four BALB/c nude mice were inoculated subcutaneously with MDA-MB-231 cells and randomly divided into four groups (16 mice per group, ten mice were used for determination of survival and six for determination of tumor volume and H&E, TUNEL, and immunohistochemistry analyses). After 5 days of inoculation**,** mice were treated with vehicle, CQ, IH, and the CQ/IH combination. **a** Comparison of the overall survival of mice between vehicle, CQ, IH and CQ/IH (*n* = 10 mice per group). Statistical significance in survival was determined by log-rank test. ^***^P < 0.01, comparison between vehicle and CQ/IH. **b** Average tumor volume in mice treated with vehicle, CQ, IH, and CQ/IH (*n* = 6 mice per group). ^***^ *P* < 0.001, comparison between vehicle and CQ/IH. **c** Body weight of mice during the 30 days of treatment. **d** Tumor tissues were sectioned and subjected to H&E, TUNEL, and immunohistochemistry analyses for determination of morphology, apoptosis, and the expression of C-Caspase 3, p-CaMKII (T286), and p-Drp1 (S616). Original magnification × 200. Scale bars: 100 μm. **e** The proposed mechanism of the synergistic interactions between isorhamnetin and chloroquine

To determine whether our in vitro findings that the inhibition of autophagy by CQ can be sensitized to IH-induced cell death could be replicated in vivo, we next examined the effect of CQ on the inhibitory efficacy of IH in vivo using a TNBC xenograft mouse model. After inoculation, mice received injections of either vehicle, CQ (40 mg/kg), IH (20 mg/kg) or a combination of these for 80 days. Kaplan–Meir survival analysis showed that the median survival time of the vehicle control group (*n* = 10) was approximately 34 days. Exposing mice only to CQ or IH resulted in mild increases in their survival time (36 or 39 days, *n* = 10). However, a combination of CQ/IH significantly improved the median survival of the mice to 62 days (*P <* 0.001 compared to the vehicle control) (Fig. 6a). We next determined the effect of the CQ/IH combination on the tumor growth of the TNBC xenografts. CQ itself had no significant impact on tumor growth and IH treatment modestly inhibited tumor growth; however, a combination of CQ/IH caused greater inhibition of tumor growth (*P <* 0.001, compared to the vehicle control) (Fig. 6b). No statistically significant changes in body weight were noted in either vehicle control, CQ, IH, or the combination (Fig. 6c).

We next examined the morphological changes, apoptosis, and CaMKII/Drp1 signaling pathway in the tumor tissue of the TNBC xenografts using H&E staining, TUNEL, and immunohistochemistry analyses. Whereas CQ itself had little effect on morphological changes and IH treatment led to modest decreases in the number of cancer cells, the combination of CQ/IH dramatically decreased the number of cancer cells and exhibited signs of the infiltration of inflammatory cells and apoptosis (Fig. 6d, top panels). The TUNEL and immunohistochemistry analyses showed modest increases in apoptosis and cleaved caspase 3 in tumor sections of mice treated with IH alone, and a significant increase in apoptosis and cleaved caspase 3 in tumor sections of mice treated with CQ/IH (Fig. 6d, second and third panels). Furthermore, combined treatment with CQ/IH led to significant increases in the interaction of CaMKII and Drp1 (Fig. 6d, fourth panel). Taken together, these findings indicate a combination of CQ/IH inhibited tumor growth and induced apoptosis TNBC xenograft in vivo through interruption of CaMKII/Drp1 signaling.

## Discussion

In this study, we provide, for the first time, compelling evidence that inhibition of autophagy/mitophagy selectively potentiates IH-induced mitochondrial fission and apoptosis in TNBC cells. Increasing evidence reveals the close relationship between autophagy and apoptosis [33–35]. Inhibition of autophagy often causes excessive autophagy (e.g. increased amounts of both LC3-II and p62). This leads to autophagic stress, and ultimately, apoptosis [33]. In the present study, we found that inhibition of autophagy by CQ potently enhanced IH-induced cell death. It is more likely that the excessive autophagosome accumulation could be involved in synergistic interactions between CQ and IH in mediating cell death based on the following observations. The first is that the combination of CQ and IH led to an increased accumulation of autophagosomes (e.g. increased amounts of both LC3-II and p62 in mitochondria and increased accumulation of mitophagosomes). Secondly, partially blocking autophagosome formation with siRNA against ATG5 markedly attenuated combination-mediated increased amounts of LC3-II in mitochondria and accumulation of mitophagosomes. The third was that the knockdown of ATG5 abrogated combination-induced PARP degradation, caspase-3 activation, cytochrome c release, and apoptosis. These findings suggest that the CQ/IH combination inducing cell death depends largely on excessive autophagy.

In particular, we demonstrated the inhibition of autophagy/mitophagy by CQ-sensitized TNBC cells to IH-induced cell death through Drp1-dependent mitochondrial fission. Drp1 is a member of the conserved dynamin GTPase superfamily, which includes a broad range of membrane fission proteins [36]. During mitochondrial fission, Drp1 is translocated from the cytosol to prospective fission sites on the mitochondria [37]. Mitochondrial fission leads to cytochrome c release and the activation of caspases, which can ultimately lead to cell death [38]. Drp1 is one of the main regulators of mitochondrial fission, and its recruitment to mitochondria is tightly regulated by posttranslational modifications such as phosphorylation, S-nitrosylation, SUMOylation, and ubiquitination [39]. Among these modifications on Drp1, phosphorylation has been most extensively studied. Drp1-dependent mitochondrial fragmentation is controlled by phosphorylation at two different conserved sites, serine 616 and 637 [40]. The two sites seem to have opposing effects on mitochondrial shape [41, 42]. While Drp1 phosphorylation at S616 promotes mitochondrial fission, Drp1 phosphorylation at S637 suppresses it [24]. In this study, we demonstrate that the phosphorylation of Drp1 at S616 and its mitochondrial translocation are essential for mitochondrial fission and apoptosis mediated by the combination of CQ/IH based on multiple lines of evidence. The first is that the combination of CQ/IH led to phosphorylation of Drp1 at S616 but did not affect phosphorylation of Drp1 at S637. The second is that the combination of CQ/IH caused the mitochondrial translocation of Drp1. Our study also showed that the combination of CQ/IH caused the mitochondrial translocation of Bax. Increasing evidence reveals that, in response to apoptotic stimuli, mitochondrial translocation of Bax is essential for mitochondrial outer membrane permeabilization (MOMP) and the ensuing release of cytochrome c [43]. Drp1 has previously been reported to be critical for cytochrome c release and apoptosis [44]. A more likely possibility is that Drp1 might interact directly with activated Bax, creating a complex that is more active in MOMP, mitochondrial fission, and cytochrome c release [45]. Consistent with this, our finding indicates that Drp1 is colocalized with Bax at mitochondrial fission sites during the combination of CQ/IH mediating cytochrome c release and apoptosis. Thus, these findings indicate that the phosphorylation of Drp1 (S616) and its mitochondrial translocation is critical for mitochondrial fission, cytochrome c release, and apoptosis in TNBC cells in response to the combination of CQ/IH.

This study also provides evidence that the activation of calmodulin-dependent protein kinase II (CaMKII) is crucial to combination-induced mitochondrial fission and apoptosis in TNBC cells. CaMKII is a multifunctional serine/threonine protein kinase that plays an important role in the transmission of Ca^2+^ signals to regulate various cellular processes [46, 47]. In a recent study, the activation of CaMKII and the subsequent phosphorylation of Drp1 at S616 are critical for mitochondrial fission during chronic β-adrenergic stimulation [26]. It has also been demonstrated that CaMKII mediates radiation-induced mitochondrial fission by regulating the phosphorylation of Drp1 at S616 [37]. Consistent with these results, the induction of mitochondrial fission and apoptosis by the combination of CQ/IH was associated with the activation of CaMKII mediating phosphorylation (S616) and the mitochondrial translocation of Drp1. Firstly, combined treatment with CQ/IH induces the phosphorylation of CaMKII (Thr286) and Drp1(S616). Secondly, mitochondrial translocation of Drp1, mitochondrial fission, and apoptosis were blocked in cells overexpressing CaMKII^T286A^ (occluding Thr286 phosphorylation) but promoted in cells overexpressing CaMKII^T286D^ (mimicking Thr286 phosphorylation) in response to the CQ/IH combination. Thirdly, the knockdown of CaMKII with siRNA significantly blocked the mitochondrial translocation of Drp1, mitochondrial fission, and apoptosis mediated by the combination of CQ/IH. Very surprisingly, we discovered that the CQ/IH combination led to the mitochondrial translocation of CaMKII. It is much more possible that the phosphorylation of CaMKII (Thr286) and its mitochondrial translocation may serve as switches which determine the phosphorylation (S616) and mitochondrial translocation of Drp1 during mitochondrial fission and apoptosis induced by the CQ/IH combination, as shown by a variety of evidence. The first is that the combination of CQ/IH led to the phosphorylation of both CaMKII (Thr286) and Drp1 (S616) and their mitochondrial translocation. Secondly, the combination of CQ/IH promoted the interaction and colocalization of CaMKII and Drp1 in the mitochondria. Thirdly, the mitochondrial translocation of CaMKII and Drp1, mitochondrial fission, and apoptosis were blocked in cells overexpressing CaMKII^T286A^ but promoted in cells overexpressing CaMKII^T286D^, in response to the combination CQ/IH. The final piece of evidence is that the knockdown of CaMKII with siRNA significantly blocked the mitochondrial translocation of CaMKII and Drp1, mitochondrial fission, and apoptosis mediated by the CQ/IH combination. To the best of our knowledge, this is the first report finding that the mitochondrial translocation of CaMKII is required for combination-mediated Drp1-dependent mitochondrial fission and cell death.

Several lines of evidence demonstrate that ROS play critical roles in CaMKII activation-mediated apoptosis [48–51]. ROS, including O_2_^•-^, H_2_O_2_, and OH·, are recognized as signaling molecules that are mobilized in response to various apoptotic stimuli [52]. In this study, we employed three antioxidants—TBAP, catalase, and sodium formate, which primarily act on O_2_^• -^, H_2_O_2_, and OH·, respectively—to investigate the involvement of individual ROS in combination-mediated mitochondrial fission, apoptosis, and perturbations in signaling events. Our results suggest that O_2_^• -^ plays an essential role in combination-mediated apoptosis in TNBC cells, based on several lines of evidence. The first is that TBAP, a O_2_^•-^ scavenger, essentially abrogated CQ/IH-mediated ROS generation in TNBC cells, whereas catalase (a H_2_O_2_ scavenger) and SF (a OH· scavenger) failed to do so. Secondly, TBAP, but not catalase and SF, noticeably prevented phosphorylation of CaMKII (Thr286) and Drp1 (S616) and their mitochondrial translocation mediated by CQ/IH. Thirdly, TBAP, but not catalase and SF, markedly attenuated colocalization of CaMKII and Drp1 at mitochondria induced by CQ/IH. Finally, TBAP, but not catalase and SF, significantly inhibited mitochondrial fission and apoptosis mediated by CQ/IH. Together, these findings suggest that O_2_^• -^ is primarily responsible not only for CQ/IH-mediated lethality in TNBC cells but also for perturbations in the CaMKII/Drp1 signaling pathway.

## Conclusions

In summary, the present findings demonstrate for the first time that the inhibition of autophagy/mitophagy by CQ enhanced IH-mediated apoptosis by triggering mitochondrial fission in TNBC cells. Our findings highlight a critical role of ROS-mediating CaMKII/Drp1 signaling in the regulation of mitochondrial fission and apoptosis induced by the combination of CQ/IH. These findings support a hypothetical model of the synergistic effects of CQ/IH (Fig. 6e). In this model, the CQ/IH combination induces production of ROS, particularly the O_2_^•-^ free radical. This, in turn, promotes the phosphorylation of CaMKII/Drp1 and their mitochondrial translocation, leading to the mitochondrial translocation of Bax; this culminated in mitochondrial fission, caspase activation, and apoptosis. Our findings also suggest that IH has the potential for further development as a novel chemotherapeutic agent, and that a combination of IH with classic autophagy/mitophagy inhibitor could represent a novel therapeutic strategy for the treatment of TNBC.

## Additional file

Additional file 1: **Figure S1.** Combined treatment with CQ/IH induces apoptosis in BT549 cells. **Figure S2.** Excessive accumulation of mitophagosomes contributes to apoptosis induced by combination of CQ/IH in MDA-MB-231 cells. **Figure S3**. Combined treatment with CQ/IH induces phosphorylation of Drp1 (Ser616) and mitochondrial translocation of Drp1 in BT549 cells. **Figure S4**. Combined treatment with CQ/IH induces the colocalization of Drp1 and Bax at mitochondria in MDA-MB-231 cells. **Figure S5**. Combined treatment with CQ/IH induces phosphorylation of CaMKII (Thr286) and mitochondrial translocation of CaMKII (Thr286) in BT549 cells. **Figure S6**. CaMKII mutation blocks mitochondrial fission and apoptosis induced by combination of CQ/IH. **Figure S7**. Knockdown of CaMKII blocks mitochondrial fission and apoptosis induced by combination of CQ/IH. **Figure S8**. Effects of antioxidants on CQ/IH-induced ROS generation, mitochondrial fission, apoptosis, and cell signaling proteins. (DOCX 4596 kb)

## Abbreviations

CaMKII: Calmodulin-dependent protein kinase II
CQ: Chloroquine
Drp1: Dynamin-related protein 1
IH: Isorhamnetin
ROS: Reactive oxygen species
TNBC: Triple-negative breast cancer
