# Supplementary material for: ROS-mediated activation and mitochondrial translocation of CaMKII contributes to Drp1-dependent mitochondrial fission and apoptosis in triple-negative breast cancer cells by isorhamnetin and chloroquine
Source: J Exp Clin Cancer Res. 2019 May 28;38:225. doi: 10.1186/s13046-019-1201-4 (PMC6540563; doi:10.1186/s13046-019-1201-4)
Supplement: Supplementary file 1 — Figure S1. Combined treatment with CQ/IH induces apoptosis in BT549 cells. Figure S2. Excessive accumulation of mitophagosomes contributes to apoptosis induced by combination of CQ/IH in MDA-MB-231 cells. Figure S3. Combined treatment with CQ/IH induces phosphorylation of Drp1 (Ser616) and mitochondrial translocation of Drp1 in BT549 cells. Figure S4. Combined treatment with CQ/IH induces the colocalization of Drp1 and Bax at mitochondria in MDA-MB-231 cells. Figure S5. Combined treatment with CQ/IH induces phosphorylation of CaMKII (Thr286) and mitochondrial translocation of CaMKII (Thr286) in BT549 cells. Figure S6. CaMKII mutation blocks mitochondrial fission and apoptosis induced by combination of CQ/IH. Figure S7. Knockdown of CaMKII blocks mitochondrial fission and apoptosis induced by combination of CQ/IH. Figure S8. Effects of antioxidants on CQ/IH-induced ROS generation, mitochondrial fission, apoptosis, and cell signaling proteins. (DOCX 4596 kb) [file 13046_2019_1201_MOESM1_ESM.docx]

**
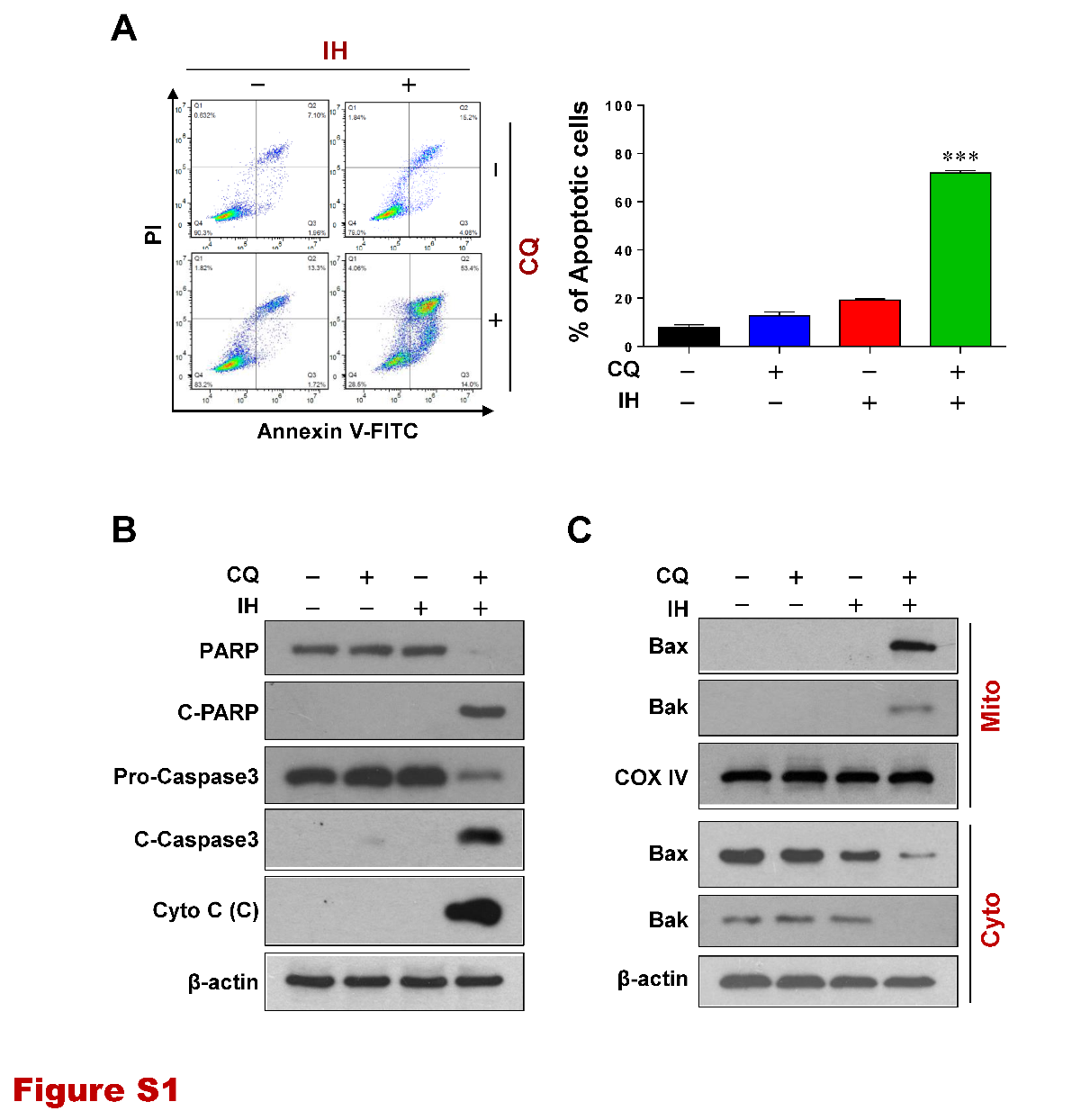
**

**Figure S1. Combined treatment with CQ/IH induces apoptosis in BT549 cells.**

(A) BT549 cells were combined treated with CQ (20 μM) and IH (10 μM) for 48 h. Apoptosis was determined by Annexin V-FITC/PI staining and flow cytometry (mean ± SD for 3 independent experiments; ^***^*P* < 0.001 compared with control or CQ and IH treatment alone). (B and C) Total cellular extract, cytosol and mitochondrial fractions were prepared and subjected to Western blot using antibodies against total PRAP, C-PARP, pro- Caspase 3, C-Caspase-3, cytochrome c (Cyto C), Bax and Bax. β-actin and COX IV were used as loading control.

**
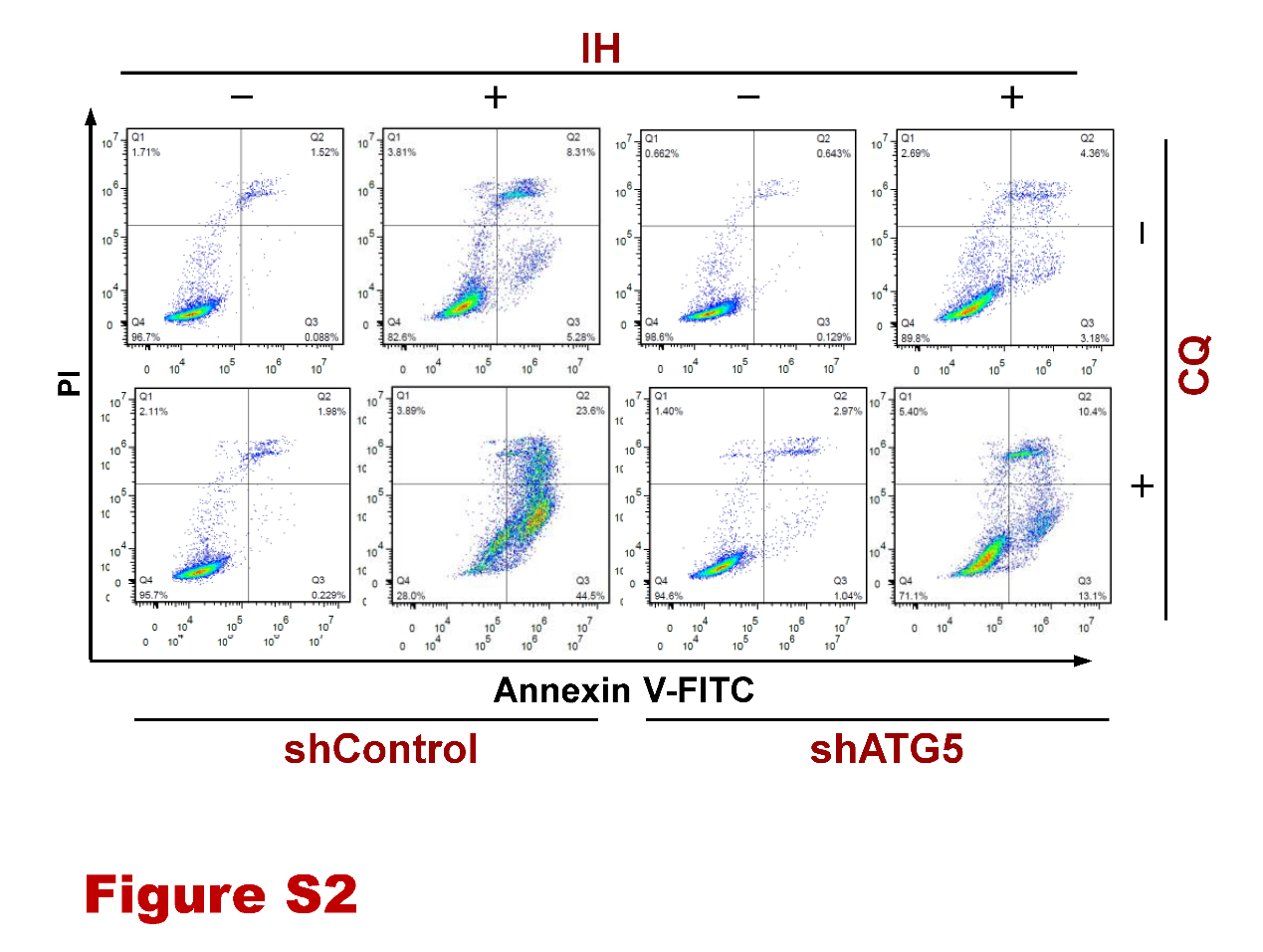
**

**Figure S2. Excessive accumulation of mitophagosomes contributes to apoptosis induced by combination of CQ/IH in MDA-MB-231 cells.**

(A) MDA-MB-231 cells stably expressing shControl or shATG5 were treated with CQ (20 μM) in presence or absence of IH (10 μM) for 48 h, Apoptosis was determined by Annexin V-FITC/PI staining and flow cytometry.


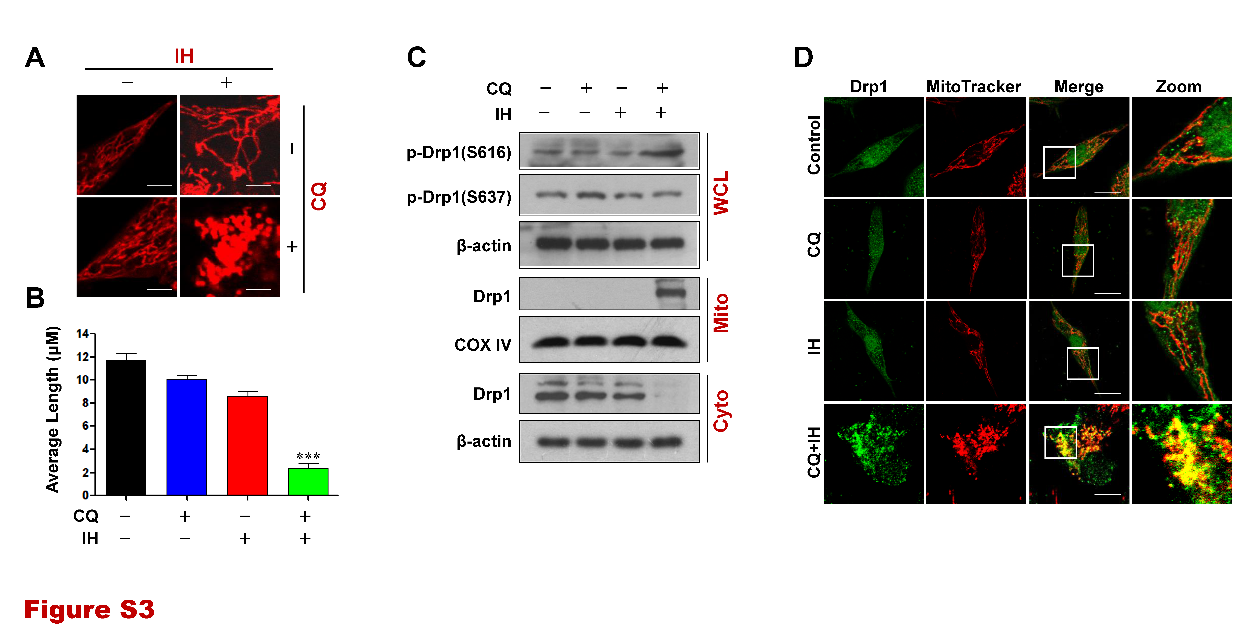


**Figure S3.** **Combined treatment with CQ/IH induces phosphorylation of Drp1 (Ser616) and mitochondrial translocation of Drp1 in BT549 cells.**

BT549 cells were treated with CQ (20 μM) in presence or absence of IH (10 μM) for 48 h. (A) Mitochondrial morphology was observed by MitoTracker Red CMXRos staining and confocal microscopy. Scale bars: 10 μm. (B) Mitochondrial length was measured with ImageJ software. 50 cells of 3 independent experiments (mean ± SD, ^***^*P* < 0.001 compared with control). (C) Whole cellular lysates (WCL) and cytosolic (Cyto)/mitochondrial (Mito) fractions were prepared and subjected to Western blot using antibodies against phospho-Drp1 (p-Drp1) (S616), p-Drp1 (S637), and Drp1. (D) The colocalization of Drp1 (green) and MitoTracker (red) was examined by confocal microscopy. Scale bars: 10 μm.

**
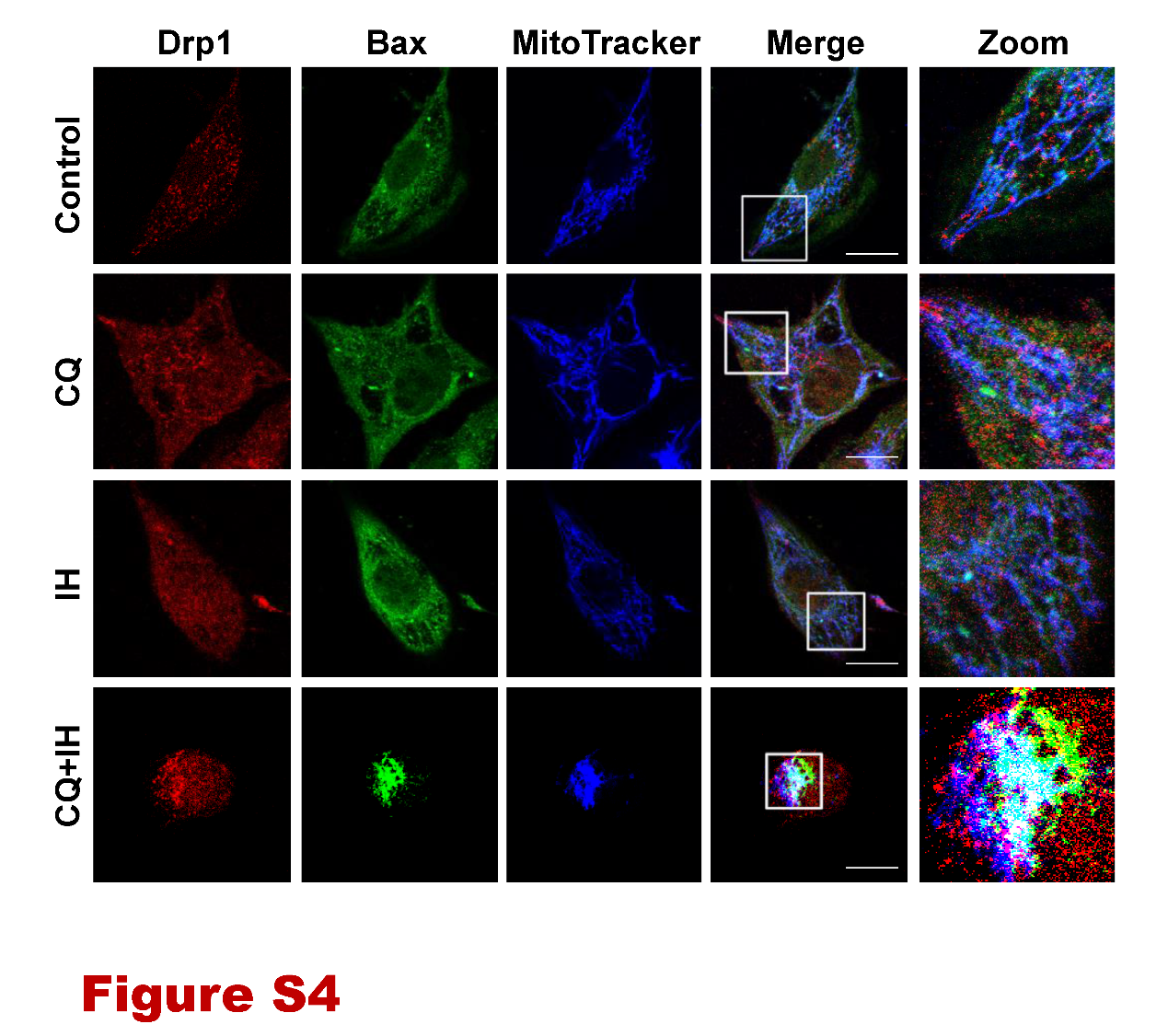
**

**Figure S4. Combined treatment with CQ/IH induces the colocalization of Drp1 and Bax at mitochondria in MDA-MB-231 cells.**

MDA-MB-231 cells cells were treated with CQ (20 μM) in presence or absence of IH (10 μM) for 48 h. The colocalization of Drp1 (red), Bax (green) and MitoTracker (blue) was examined by confocal microscopy. Scale bars: 10 μm.

**
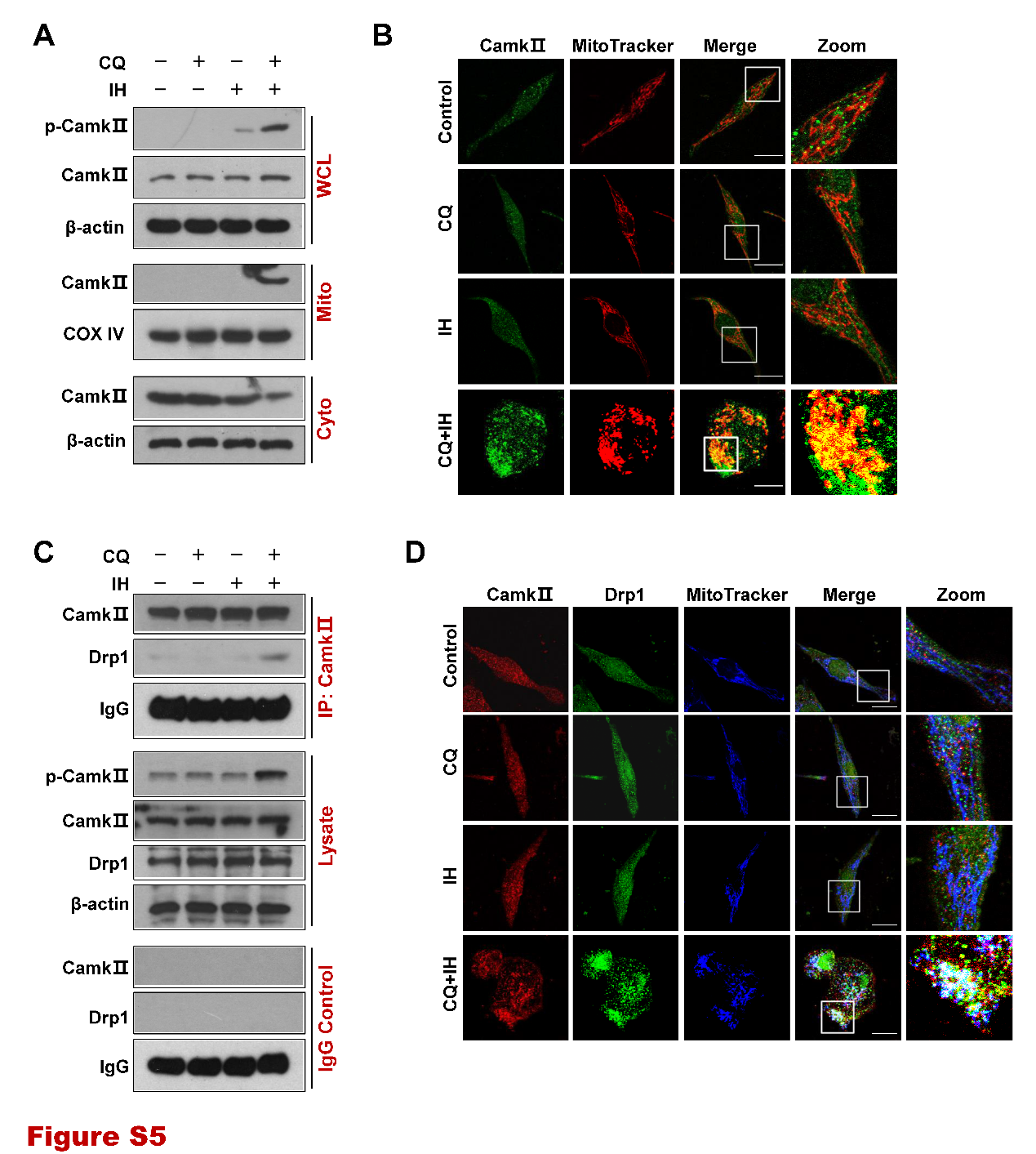
**

**Figure S5. Combined treatment with CQ/IH induces phosphorylation of CaMKⅡ (Thr286) and mitochondrial translocation of CaMKⅡ (Thr286) in BT549 cells.**

BT549 cells were treated with CQ (20 μM) in presence or absence of IH (10 μM) for 48 h. (A) The expression of p-CaMKII and CaMKII in WCL, Cyto, or Mito was examined by Western blot. (B) The colocalization of CaMKII (green) and MitoTracker (red) was examined by confocal microscopy. Scale bars: 10 μm. (C) Whole cell lysates were prepared and subjected to immunoprecipitation using anti-CaMKII, and the associated CaMKII and Drp1 were determined using immunoblotting. (D) The colocalization of CaMKII (red), Drp1 (green), and MitoTracker (blue) was examined by confocal microscopy. Scale bars: 10 μm.

**
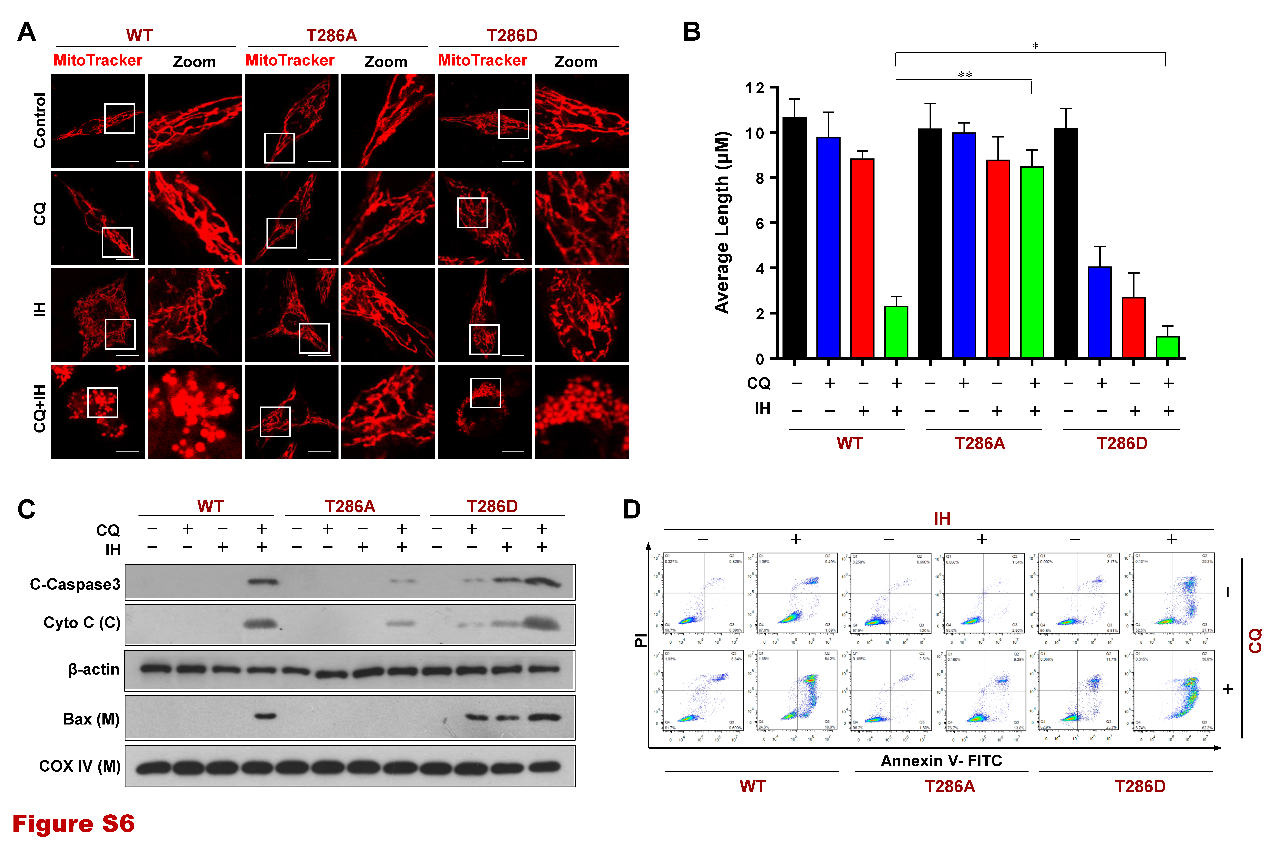
**

**Figure S6. CaMKII mutation blocks mitochondrial fission and apoptosis induced by combination of CQ/IH.**

MDA-MB-231 cells transfected with either wild type CaMKII (WT), mutant CaMKII^T286A^ (T286A) or mutant of CaMKII^T286D^ (T286D) were treated with CQ (20 μM) in presence or absence of IH (10 μM) for 48 h. (A) Mitochondrial morphology was observed by MitoTracker Red CMXRos staining and confocal microscopy. Scale bars: 10 μm. (B) Mitochondrial length was measured with ImageJ software. 50 cells of 3 independent experiments (mean ± SD, ^*^*P* < 0.05 or ^**^*P* < 0.01). (C) Total cellular extract, cytosol and mitochondrial fractions were prepared and subjected to western blot using antibodies against cleaved caspase-3, cytochrome c (Cyto C), and Bax. β-actin and COX IV were used as loading control. (D) Apoptosis was determined by Annexin V-FITC/PI staining and flow cytometry.


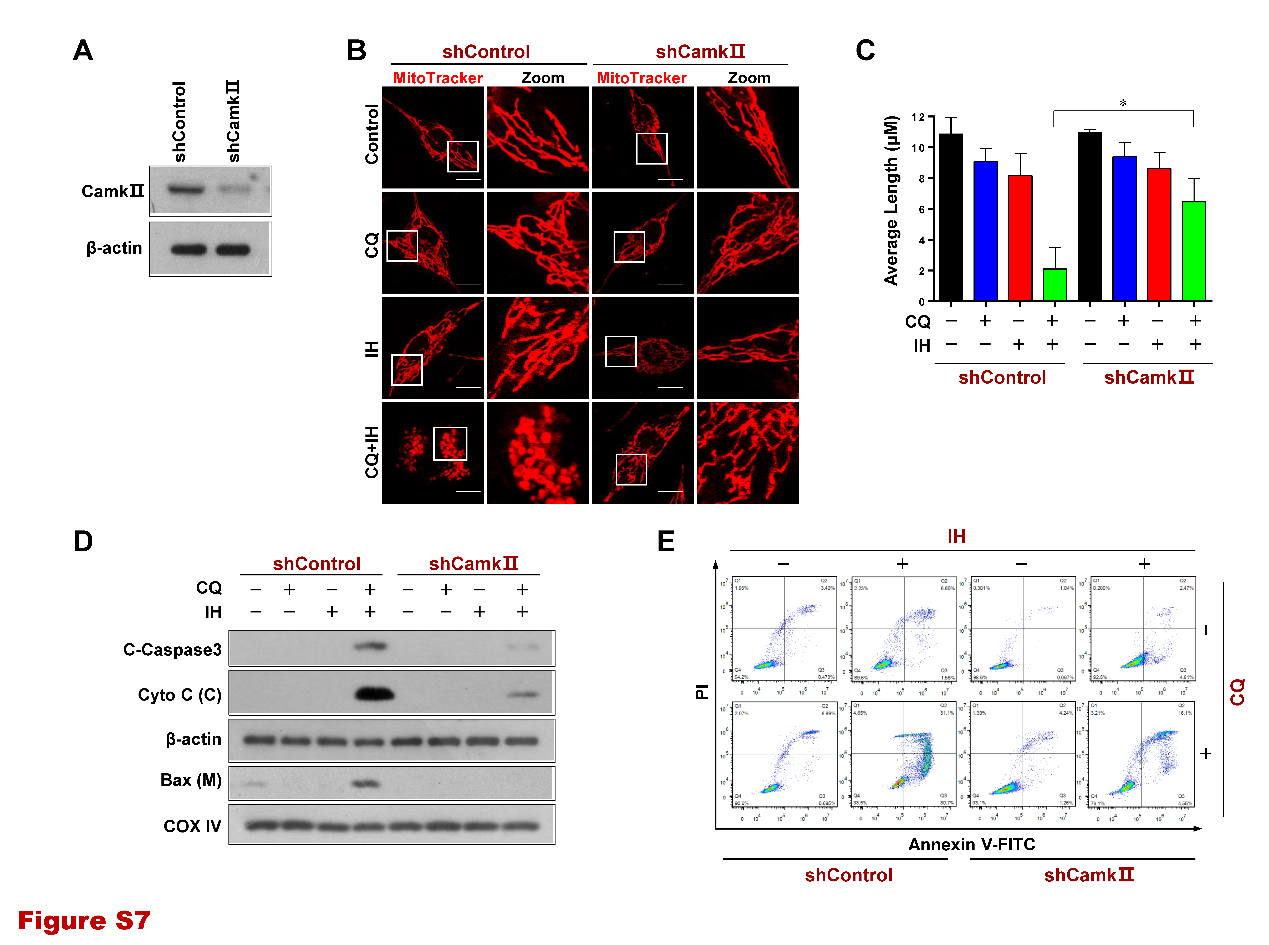


**Figure S7. Knockdown of CaMKII blocks mitochondrial fission and apoptosis induced by combination of CQ/IH.**

(A) Cells were transfected with control shRNA (shControl) or sh CaMKII, and Western blot analysis was used to determine the expression of CaMKII. For B-E, Cells stably expressing shControl or sh CaMKII were treated with CQ (20 μM) in presence or absence of IH (10 μM) for 48 h. (B) Mitochondrial morphology was observed by MitoTracker Red CMXRos staining and confocal microscopy. Scale bars: 10 μm. (C) Mitochondrial length was measured with ImageJ software. 50 cells of 3 independent experiments (mean ± SD, ^*^*P* < 0.05). (D) Total cellular extract, cytosol and mitochondrial fractions were prepared and subjected to western blot using antibodies against cleaved caspase-3, cytochrome c (Cyto C), and Bax. β-actin and COX IV were used as loading control. (E) Apoptosis was determined by Annexin V-FITC/PI staining and flow cytometry.


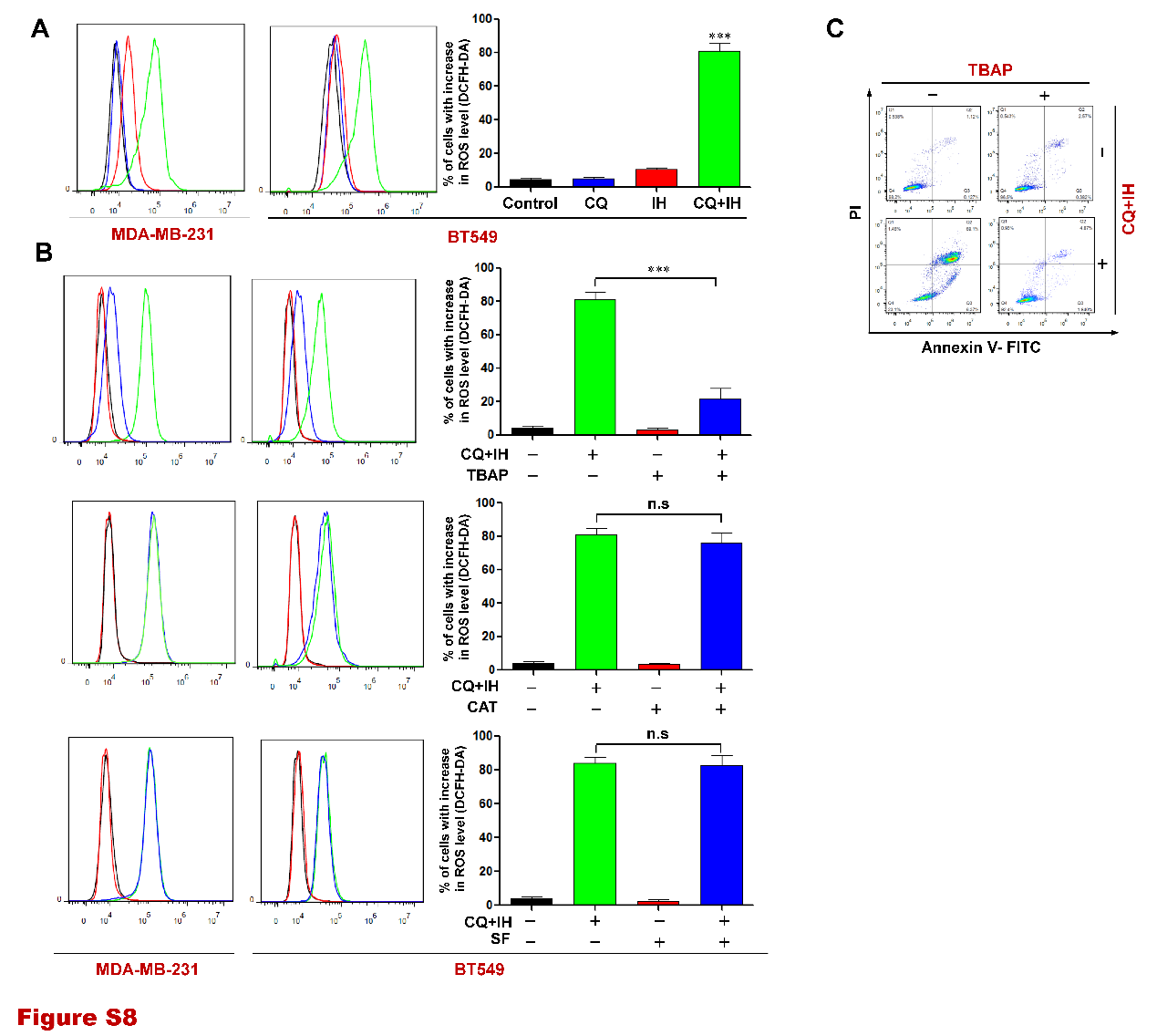


**Figure S8. Effects of antioxidants on CQ/IH-induced ROS generation, mitochondrial fission, apoptosis, and cell signaling proteins.**

(A) MDA-MB-231 and BT549 cells were treated with CQ (20 μM) in presence or absence of IH (10 μM) for 6 h. Cells were stained with DCFHDA, and ROS production was analyzed by flow cytometry, The values represent the mean ± SD for 3 separate experiments (^***^*P* < 0.001 compared with control or CQ and IH treatment alone). (B) Cells were pretreated with antioxidants including as TBAP (200 μM), catalase (5000 U/ml), and sodium formate (SF, 2mM) for 1 h, followed by combined treatment with CQ/IH, after which cells were stained with DCFHDA, and ROS production was analyzed by flow cytometry, The values represent the mean ± SD for 3 separate experiments (^***^*P* < 0.001 or n.s compared with TBAP/CQ/IH, CAT/CQ/IH or SF/CQ/IH). (C) Cells were pretreated with TBAP, followed by combination of CQ/IH, Apoptosis was determined by Annexin V-FITC/PI staining and flow cytometry.
